# Natural Language Processing and Machine Learning Techniques for Analyzing Conversations About Nutritional Yeasts in the United States and France: Retrospective Social Media Listening Study

**DOI:** 10.2196/60528

**Published:** 2025-05-01

**Authors:** Jean-François Jeanne, Joelle Malaab, Antoine Vanhove, Florian Mourey, Manissa Talmatkadi, Stéphane Schück

**Affiliations:** 1 Gnosis by Lesaffre, Lesaffre Group, Marcq-en-Baroeul, France; 2 Kap Code, 4 rue de Cléry, Paris, 75002, France, 33 01 44 82 74 74

**Keywords:** nutritional yeast, social media listening, natural language processing, machine learning, infodemiology study

## Abstract

**Background:**

Nutritional yeast, an inactive form of *Saccharomyces cerevisiae*, has recently become increasingly popular as a food supplement and healthy ingredient, especially among individuals following plant-based diets. It is valued for its health benefits and high content of B vitamins, minerals, and protein. Social media has enabled people to share information and personal experiences at an unprecedented level, further amplifying conversations around health and nutrition. With the rise of social media, data mining techniques like natural language processing and machine learning are increasingly used for analyzing the large amounts of information generated on these platforms.

**Objective:**

This study aimed to analyze social media data from the United States and France to identify the most frequently discussed topics among nutritional yeast consumers. The objective was to fill gaps in our understanding of the perceptions, experiences, and usage trends related to nutritional yeast.

**Methods:**

This study was retrospective, using social media data geolocated in the United States and France, posted by users discussing nutritional yeast between December 2017 and September 2023. Data cleaning and filtering were done using natural language processing methods and specific algorithms. Biterm topic modeling was applied to identify the most frequently discussed topics.

**Results:**

A total of 36,642 posts written by 28,069 users discussing nutritional yeast were identified across 1039 publicly available online sources. This included 34,292 posts from the United States (26,154 users across 994 sources) and 2350 from France (n=1915 users across 45 sources). Twitter was the most commonly used platform in both countries, accounting for 39.6% of posts in the United States (13,587/34,292) and 84.3% in France (1982/2350). In the United States, conversations centered around the role of nutritional yeast role as a vegan nutrient source (n=12,345, 36.0%). Several users highlighted its culinary versatility as a natural seasoning (n=8093, 23.6%) and its health and skin benefits (n=6173, 18.0%). In France, discussions frequently focused on nutritional yeast’s use in dietary supplement routines in various forms (n=1177, 50.1%), emphasizing its benefits alongside other supplements such as castor oil, particularly noted for effects on nails and hair (n=928, 39.5%).

**Conclusions:**

This social media listening study identified the perceptions and preferences of nutritional yeast users in France and the United States. Researchers and health care professionals can reflect on these findings to investigate the potential health benefits of nutritional yeast for specific groups and its long-term impact on different diets and lifestyles. Marketers may also use this information to create customized strategies that better align with the preferences and needs of each market.

## Introduction

For thousands of years, yeast has been used in the preparation of food and beverages due to its fermentation properties in baking and brewing. Nutritional yeast, an inactive form of *Saccharomyces cerevisiae* [1], has been consumed for its health benefits since the early 20th century. More recently, it has become popular as a food supplement and cooking ingredient. Nutritional yeast—also known as brewer’s yeast—is rich in B vitamins, minerals, and protein, making it both nutritious and flavorful [2,3]. In today’s world, where healthy living is a priority for many, it has become an important part of vegan and vegetarian diets. Individuals following a raw vegan diet and experiencing B12 deficiency showed significant improvement after supplementing with nutritional yeast and sublingual B12 tablets, whereas probiotic supplements did not yield similar benefits [4]. Additionally, a randomized double-blind clinical trial involving adults with type 2 diabetes mellitus found that brewer’s yeast supplementation improved blood pressure [5]. Another study reported beneficial effects on serum triacylglycerol and glucose tolerance [6]. Nutritional yeast has also been shown to support the immune and gastrointestinal systems; it includes probiotics and postbiotics that help in immune modulation, strengthening the body’s defense against pathogens [7,8]. Furthermore, it contributes to the balance and growth of gut microbiota, promoting gastrointestinal health and overall well-being [9,10]. As awareness of healthy nutrition continues to grow, nutritional yeast is gaining popularity among a broader population looking for healthier food options or to modify their shopping, cooking, and eating habits as a whole [3,11].

As consumers become more health conscious and search for information about food supplements, they are increasingly turning to social media to gain knowledge about health and nutrition [12]. Users engage online for various purposes, including sharing information and personal experiences, participating in medical education [13], gaining awareness around health campaigns [14,15], and becoming members of online communities [16]. Twitter, Facebook, and health-related forums are increasingly used for information about nutrition, dietary supplements, and related topics [17-19], especially among younger individuals [20].

Given the vast volume of data generated through social media, data mining is being applied to analyze user-generated content [21]. This artificial intelligence technique includes natural language processing, which allows machines to analyze textual data to comprehend human language [22], and machine learning, which creates algorithms that recognize patterns in data and produce predictions [23], both of which are progressively used within this framework [24]. As a result, public perceptions, trends, and behaviors can be identified.

Although nutritional yeast is gaining popularity, knowledge about public opinions and usage patterns is still limited. While previous research has explored its health benefits, little is known about the discussions surrounding it, particularly on social media. Moreover, comparative analyses between different cultural and geographical contexts, such as the United States and France, are scarce. This study, which focuses on online discussions in the United States and France, aims to fill this gap by exploring how nutritional yeast is discussed, used, and perceived. Through data mining, we aim to identify the main topics of discussion on social media among consumers of nutritional yeast.

## Methods

### Study Design and Population

The present study is retrospective, using data from social media posts by nutritional yeast users geolocated in the United States and France.

#### Data Extraction

Between December 2017 and September 2023, messages were retrieved from general, publicly accessed sites (eg, Twitter, Reddit) and health-related forums (eg, Doctissimo in France and HealthUnlocked [25] in the United States). Due to restricted data access and closed groups, Facebook and WhatsApp were excluded from this study. An extraction query featuring relevant keywords was first developed to identify pertinent messages. Keywords associated with nutritional yeast were included in English (eg, brewer’s yeast, nutritional yeast) and French (eg, levure de bière, *Saccharomyces cerevisiae*). The complete list of keywords was subsequently used in the extraction query (Multimedia Appendix 1).

Using the Brandwatch extractor (Cision Ltd.) [26], we identified and gathered all publicly available posts that contained one of the required keywords, along with their associated metadata (eg, author, publication date). Posts were also geolocated using Brandwatch. When applicable, various spellings of a keyword were considered in the extraction query. For example, the word bière was inserted as biere, bierre, and bier and the words brewer’s yeast were included as brewer’s yeast and brewer yeast. This approach allowed us to increase exhaustivity by including the various ways an internet user might spell a keyword.

#### Data Cleaning

First, messages were harmonized to ensure consistency across the dataset. We switched all characters in the messages to lowercase format and removed all accents and apostrophes from words; this approach helped achieve a smoother cleaning, and eliminating duplicates.

The cleaning process then established a list of exclusion criteria, removing messages from sources deemed unsafe or irrelevant (eg, advertising websites, forums related to cars, pets, or animals), duplicate posts, messages containing five words or less, and posts exceeding 10,000 characters. Generally, a message with fewer than five words does not contain enough information to be effectively exploitable and interpreted. Messages exceeding 10,000 characters are rare and nevertheless, are excluded from the analysis dataset due to excessive processing time.

To determine the number of messages for each keyword, we applied a “presence” step. This involved automatically searching the dataset for keywords and identifying the messages that contained them. Not all messages mentioning nutritional yeast were from people who had consumed it—some may have been discussing it without personal use. To address this, we applied a supervised machine learning algorithm [27] to identify messages specifically from nutritional yeast users. This algorithm classified each message by determining whether the user had taken the mentioned “treatment” (nutritional yeast), assigning a value of 0 for no intake and 1 for intake. It analyzed language to make predictions, including first-person pronouns to detect personal use, sentiment words to understand the emotional context, verb tenses for timing, negation words, and possessive pronouns to detect whether a statement denied or confirmed use. The algorithm was trained and tested on a sample of 1563 messages that spanned a range of pathologies. Its ability to detect whether a treatment was taken generated the following performance results: accuracy, 72%; *F*_1_-score, 74%; sensibility, 78%; specificity, 66%; and precision, 70%. To extend its application to English-language messages, we translated these into French before applying the algorithm, which was initially developed and optimized for French. We then separated the messages into French and US datasets based on the language of the original messages.

Multiple human annotators annotated random samples of messages to further validate the algorithm’s output. This step ensured that the algorithm’s results were pertinent and accurate.

### Topics of Discussion

The main discussion themes among nutritional yeast consumers were identified using Biterm Topic Modeling (BTM). BTM is a natural language processing approach that analyzes large volumes of text and clusters similar text based on common topics [28]. BTM automatically groups messages into different categories—each representing a specific topic—in descending order of frequency. For each category, BTM provides a list of the most recurring words, which helps understand the general focus of each topic. For example, a BTM result about breast cancer may include:

Topic 1│ Proportion of messages = x │List of most frequently mentioned words: body, image, confidence, scars, mastectomy, hair loss, appearance, self-esteem, femininity.

These words suggest a focus on body image issues, which can be further validated when reviewing the messages associated with Topic 1. This allows us to assign a title to the topic, such as *Impact of breast cancer on body image.*

In this study, we applied BTM separately to the US and French datasets without prior knowledge of the topics. For each country, BTM generated distinct categories, providing a list of the most recurrent keywords and the messages associated with each category. Based on the list of keywords, we obtained an initial understanding of each topic. We then reviewed the messages in each category to validate and refine our understanding. This allowed us to assign appropriate titles to the topics.

### Ethical Considerations

This study used only data from publicly available sources, excluding private groups, forums, and web pages. Given that users posting on public platforms automatically agree to the reuse of their information, we did not seek formal consent for this study. The findings are reported in aggregate, without personally identifiable details such as names, usernames, specific locations, or sensitive information, were deliberately removed.

## Results

### Population and Posts

**Figure 1.**
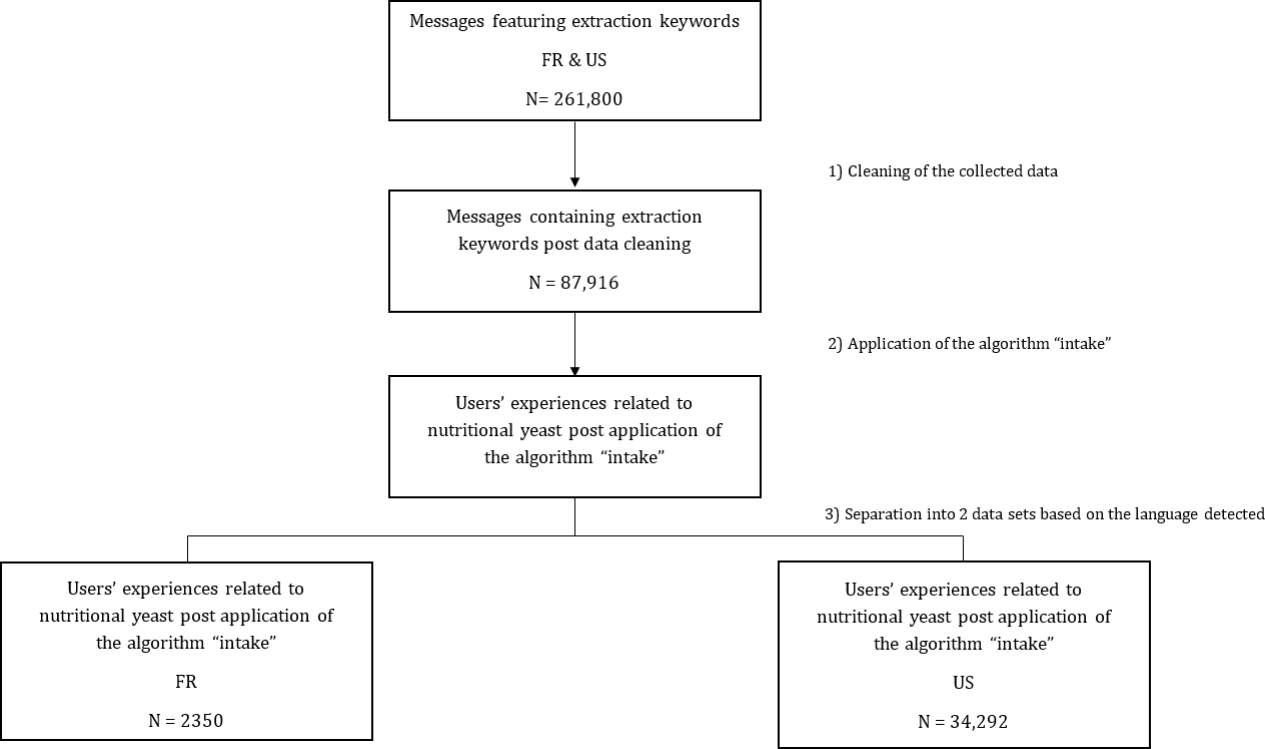
Flowchart of the data cleaning and sample selection processes showing the number of messages (N) and users discussing nutritional yeast in the United States and France between 2017-2023.

A total of 261,800 posts were initially retrieved, written by internet users discussing nutritional yeast in the United States and France. Data cleaning and processing allowed us to identify internet users who had consumed nutritional yeast. As a result, the analysis dataset contained a total of 34,292 posts in the United States written by 26,154 users and 2350 posts in France written by 1915 users (Figure 1).

### Data Sources and Temporal Evolution

Posts originated from 995 social media platforms in the United States and 45 in France. In the United States, Twitter was the main source of data (13,587/34,292; 39.6%) of posts, followed by Reddit.com (n=5333; 15.6%), and Instagram.com (n=4398, 12.8%). In France, Twitter was also the main source of data (1,982/2,350; 84.3%), followed by jeux vidéo.com (125; 5.3%) and babycenter.fr (n=93; 4.0%) (Table 1). The complete list of sources is found in Multimedia Appendix 2.

**Figure 2.**
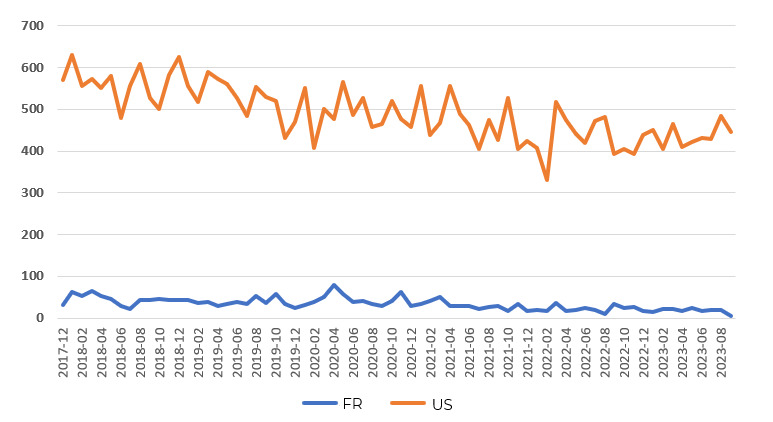
Temporal trend in the number of posts on nutritional yeast extracted between December 2017 and September 2023 from social media geolocated in the United States and France.

During the analysis period, the number of posts was higher in the United States (n=34,292) than in France (n=2350). Figure 2 shows the temporal evolution of the posts extracted in United States and France.

**Table 1.** Top 10 geolocated data sources in the United States and France for messages about nutritional yeast posted between 2017-2023 .

Forum/Social media	Posts, n (%)
United States	
Twitter	13,587 (39.6)
Reddit.com	5333 (15.6)
Instagram.com	4398 (12.8)
Whattoexpect.com	1134 (3.3)
Myproana.com	819 (2.4)
Babycenter.com	773 (2.3)
4channel.org	649 (1.9)
Myfitnesspal.com	483 (1.4)
Edsupportforum.com	307 (0.9)
Shroomery.org	276 (0.8)
France	
Twitter	1982 (84.3)
Jeux vidéos.com	125 (5.3)
Babycenter.fr	93 (4.0)
Instagram.com	32 (1.4)
Sports-sante.com	16 (0.7)
Hardware.fr	15 (0.6)
Au Feminin	14 (0.6)
Beauté test	7 (0.3)
Madmoizelle.com	5 (0.2)
Magic maman	5 (0.2)

### Topics of Discussion

After applying the BTM, various discussion topics were identified through human interpretation of the most associated terms. It is worth noting that a single message can contain multiple topics. The main revealed topics are shown in Table 2.

**Table 2.** Proportions of messages featuring the most frequently discussed topics.

Topics	Posts, n (%)
United States	
Vegan vitamin source	12,345 (36.0)
Seasoning for various recipes	8093 (23.6)
Health and Skin benefits	6173 (18.0)
Taste of cheese	4218 (12.3)
Fermented products	2160 (6.3)
Protein and calorie balance	1269 (3.7%)
France	
Dietary supplement regimens in various forms	1177 (50.1)
Castor oil for nails and hair	928 (39.5)
Personal experience, diet, and taste	223 (9.5)
Reduction of hair loss, effectiveness, and duration	115 (4.9)
Yeast, a dietary supplement rich in vitamins	96 (4.1)
Organic products (purchase and diet)	31 (1.3%)

#### Main Topics of Discussion in the United States

The most discussed theme in the United States was nutritional yeast as a vegan vitamin source (12,345/34,292; 36.0%). Users from the vegan community mentioned it as a source of vitamins, particularly B vitamins including B12—a nutrient that is challenging to obtain from a diet lacking animal products. Users also shared their experiences of how to incorporate nutritional yeast into their daily lives, such as sprinkling it on meals or blending it into smoothies. An example message reads:

*Yesterday’s lunch was a salty (and spicy ... Def went too far on the red bell pepper* 😳*) and hit the spot! Nutritional yeast may sound a bit odd, but it’s a great source of B vitamins for eaters* (translation to French in Multimedia Appendix 3)

Additionally, online discussions revealed that cooking enthusiasts have used nutritional yeast as a natural and nutritious seasoning (n=8093; 23.6%). Users posted their recipes using it as a flavor enhancer in pasta and casseroles, sharing that it added taste and creaminess without the need for dairy products. For example:

*I bought a big carton of egg whites, what I did with the tofu scrabble was to add nutritional yeast, turmeric for color and black pepper, which makes it taste whole and with whole egg and egg white too*. (translation to French in Multimedia Appendix 3*)*

Users frequently mentioned the health and skin benefits of nutritional yeast (n=6173; 18.0%) messages, with users highlighting its positive effects on skin health, particularly in managing fungal infections. They also discussed its ability to strengthen nails and improve hair health.

Nutritional yeast was also used as a cheese alternative (n=4218 messages; 12.3%). It was especially popular among vegans and individuals with lactose-intolerant, who praised it as a nutritious, plant-based option that can add flavor to dairy-free diets. Several users described it as both palatable and versatile, therefore important to include in one’s balanced diet.

Another topic of discussion was the use of nutritional yeast in home fermentation processes (n=2160; 6.3% messages) such as brewing beer, making bread, and other fermented products. However, since nutritional yeast is an inactive form of *Saccharomyces cerevisiae*, it lacks the enzymes required for fermentation. This means that the conversations discussing fermentation have mistakenly mentioned the use of nutritional yeast.

The high protein content and low-calorie count also made nutritional yeast appealing within fitness and wellness communities (n=1269; 3.7%) messages. Users noted its importance as a complete protein, ie, containing all nine essential amino acids, which is beneficial for muscle development and maintenance.

#### Main Topics of Discussion in France

In France, internet users primarily discussed the use of nutritional yeast in food supplement routines (1177/2350; 50.1%). Users mentioned how they included brewer’s yeast into their routines, sharing the different ways they consumed it in the form of capsules or pills. They reported using it for overall health or for specific concerns like hair loss and mood improvement. One user described their experience with brewer’s yeast in the following message:

*Personally, brewer’s yeast worked really well, no side effects, I also tried Oenobiol, which wasn’t bad, and now I’m starting Forcapil, apparently it’s a wonder. I can’t wait to have Rapunzel’s hair* (*translated from French, in Multimedia Appendix 3*).

Another frequently discussed topic was the use of castor oil for improving nail and hair health (n=928; 39.5%). Many users paired castor oil with brewer’s yeast, highlighting their combined benefits in strengthening and stimulating the growth of hair and nails. Users also shared specific routines, such as applying castor oil directly to their hair or nails and taking brewer’s yeast tablets as supplements. An example of a message is shown below:

*I used to lose handfuls of them too, that’s why they’re so damaged, you need to make frequent oil masks that you leave on overnight, rinse them with cold water and take brewer’s yeast to strengthen them* ☺️ (*translated from French, in Multimedia Appendix 3*).

Discussions also focused on personal experience, diet, and sensory attributes of brewer’s yeast (n=223; 9.5%). Some users described its texture and taste, while others shared their recipes and methods for incorporating brewer’s yeast into their diets. Feedback regarding its taste was generally positive, as shown in the following message:

*No problem with taking care of oneself with all kinds of oils (jojoba, coconut, castor) natural shampoo like Liperol, and brewer’s yeast in capsules and powder form (I like the taste*) (*translated from French, in Multimedia Appendix 3*).

Other messages revolved around the effect of nutritional yeast in reducing hair loss (n=115; 4.9%), as well as its role as a food supplement rich in vitamins (n=96; 4.1%), particularly the B complex. Although less commonly discussed, some users expressed a preference for organic products (n=31; 1.3%), specifically organic brewer’s yeast.

## Discussion

### Principal Findings

The objective of the study was to identify the predominant themes in conversations among internet users discussing nutritional yeast.

A total of 36,642 messages posted by 28,069 users were included in this study. Twitter emerged as the main source of discussion in both countries, accounting for 39.6% of the US dataset with 13,487 posts and 84.3% of the French dataset with 1982 posts. Additional key platforms included Reddit and Instagram in the United States, and jeux vidéo.com and babycenter.fr in France. In the US, discussions mainly focused on its role as a nutrient source for individuals following a vegan diet (36.0% of posts). Many users reported using it as a natural seasoning (23.6%) and for its benefits for health and skin (18.0%). Users also discussed cheese alternatives (12.3%), specifically using nutritional yeast as a substitute for cheese. In France, nutritional yeast was part of supplement regimens in various forms (50.1% of posts) and was used with other supplements such as castor oil, mainly to improve nail and hair health (39.5%). Additional themes included personal experiences regarding dietary habits and flavor preferences (9.5%), the role of nutritional yeast in preventing hair loss (4.9%), and its high vitamin content (4.1%).

Our findings are consistent with previous studies highlighting the role of plant-based foods in today’s diets. In the United States, nutritional yeast was already recognized as an important component in vegan nutrition, with a rapid transition to plant-based diets. In fact, veganism in the United States increased by 600% between 2014 and 2018 [29], motivated by health concerns, environmental sustainability, ethical considerations regarding animal welfare, and media influence [29-31]. Additionally, the plant-based foods market increased by 29% from 2017 to 2019 [32], highlighting the rising demand for plant-based foods among American consumers [33].

Conversely, nutritional yeast was mostly considered a dietary supplement in France, which is consistent with the French tendency to favor holistic health solutions. According to data from the Second Individual and National Study on Food Consumption (INCA), 22% of French adults consume dietary supplements on a regular basis [34], especially women, individuals aged 18 to 44, and those with higher education levels [34]. Although most adults still buy supplements from pharmacies, online purchases have significantly increased from 1% in 2015 to 11% in 2019—a clear shift in consumer habits [34]. In 2022, more than two-thirds of the French population had used dietary supplements at least once, with 32% reporting use in the past three months [35]. The market for nutritional supplements has grown to €2.6 billion and had a+3% yearly growth rate between 2021 and 2022 [36].

Our results align with previous research emphasizing the value of social media in understanding health and nutrition. With the help of data mining, social media has become a powerful source of real-world data, providing insights that traditional research methods such as clinical trials may fail to obtain. It also bypasses some of the limitations of conventional research such as lengthy timelines and complicated participant recruitment processes, making it a useful complementary tool for health research [37-39]. Infodemiological studies allow for the identification of various aspects, including specific populations, their discussion topics, the impact of health on their quality of life, as well as user perceptions and challenges [40-43]. They offer significant data, with 5.35 billion internet users and 5.04 billion actively using social media platforms in 2024 [44]. Consistent with our findings, previous studies have also identified Twitter as an essential source of data and a valuable platform for health-related discussions [38,45-48].

This study may help shape marketing strategies for each country’s unique preferences. In France, where consumers were focused on improving their overall health and well-being, marketing strategies could emphasize the holistic benefits of nutritional yeast—perhaps including it as part of multisupplement regimens. In the United States, the growing population of health-conscious, plant-based consumers presents an opportunity to focus on nutritional yeast’s role in vegan diets and its versatility in cooking, particularly as a natural flavor enhancer. Strategies could include creating educational content, collaborating with culinary experts, and launching targeted social media campaigns to effectively promote nutritional yeast’s benefits in both regions.

### Limitations

We recognize several limitations to our study. Our analysis only included openly accessible online sources; private sources such as WhatsApp, private forums, or invitation-only groups were excluded. Furthermore, the level of detail that we obtained and our understanding of the messages’ context depended on the information shared by users. Our study included a potential recall bias, as it was based on users’ self-reported data, their memory, and subjective interpretation. Additionally, individuals posting on social media may represent certain socioeconomic backgrounds and literacy capacities, which could affect the representativeness of our findings. It is also possible that relevant discussions were incorrectly removed during data cleaning. Another limitation is the variation in the number of users from each country, which may affect data representation. Additionally, since not all social media posts include geolocation data, accurately determining their country of origin can be challenging.

Despite these limitations, this study provides valuable insights into the discussions and perceptions about nutritional yeast.

### Conclusions

Nutritional yeast is a natural ingredient valued for its health benefits and culinary versatility by users in France and the United States. Social media allowed us to gain insights into consumer perspectives, experiences, and usage trends related to nutritional yeast. It also allowed the identification of the unique preferences of each country, providing more information about the health-focused French consumers and the growing American vegan population. Future research could include clinical studies to better understand the health benefits of nutritional yeast for specific groups, such as vegans or people interested in natural beauty solutions. Studies could also explore its long-term effects on various diets and wellness habits. Additionally, marketing strategies could be improved by creating tailored communication and messaging that connect more effectively with the preferences and needs of various consumer groups.

## Supplementary material

10.2196/60528 Multimedia Appendix 1 Extraction query used for Brandwatch.

10.2196/60528 Multimedia Appendix 2 List of sources.

10.2196/60528 Multimedia Appendix 3 Examples of messages in English and French.

## Abbreviations

BTM: Biterm Topic Modeling
