# Supplementary material for: Natural Language Processing and Machine Learning Techniques for Analyzing Conversations About Nutritional Yeasts in the United States and France: Retrospective Social Media Listening Study
Source: JMIR Infodemiology. 2025 May 1;5:e60528. doi: 10.2196/60528 (PMC12061346; doi:10.2196/60528)
Supplement: Multimedia Appendix 1 [file infodemiology-v5-e60528-s001.docx]

**Multimedia Appendix 1: Extraction query used for Brandwatch:**

FR extraction query :(("Levure de biere" OR "Levure de bierre" OR "Levure de bier" OR "Saccharomyces cerevisiae" OR (levure AND ((juvamine OR gayelord OR alline OR solgar OR "abbaye de sept fons" OR aroma-zone OR aromazone OR luxeol OR superdiet OR nutrimea OR vitavea OR arkopharma OR gerble OR markal OR primeal) OR beaute OR cheveu* OR peau OR ongle* OR bienetre OR "bien-etre" OR "bien etre" OR "sante mentale" OR sante OR humeur OR nutritionnel* OR fonctionnel* OR zinc OR selenium OR vitb OR niacine OR ((vit OR vitamine OR vitamines) NEAR/2 (B1 OR b2 OR b5 OR b6 OR b8 OR b9 OR b12 OR b)))))

US extraction query: ("brewer's yeast" OR "nooch" OR "nutritional yeast" OR "brewers yeast" OR "brewer yeast" OR "Saccharomyces cerevisiae" OR (yeast AND (("now foods" OR nutrilite OR "nature aids" OR "nature made" OR glorybee OR "bob's red mill" OR bragg OR kevala OR "red star") OR hair OR nail* OR skin OR beauty OR mood OR "mental health" OR health OR "mental-health" OR "wellness" OR nutritional OR functional OR zinc OR selenium OR vitb OR niacin OR ((vit OR vitamin OR vitamins) NEAR/2 (B1 OR b2 OR b5 OR b6 OR b8 OR b9 OR b12 OR b))))).
