# Supplementary material for: Natural Language Processing and Machine Learning Techniques for Analyzing Conversations About Nutritional Yeasts in the United States and France: Retrospective Social Media Listening Study
Source: JMIR Infodemiology. 2025 May 1;5:e60528. doi: 10.2196/60528 (PMC12061346; doi:10.2196/60528)
Supplement: Multimedia Appendix 2 [file infodemiology-v5-e60528-s002.docx]

**Multimedia Appendix 3: List of sources**

| **Forum/Social Media** | **n** | **Forum/Social Media** | **n** |
| --- | --- | --- | --- |
| **United States** | | **France** | |
| twitter | 13587 | twitter | 1982 |
| reddit | 5333 | jeux video | 125 |
| instagram.com | 4398 | babycenter.fr | 93 |
| whattoexpect.com | 1134 | instagram.com | 32 |
| myproana.com | 819 | sports-sante.com | 16 |
| babycenter.com | 773 | hardware.fr | 15 |
| 4channel.org | 649 | Au Feminin | 14 |
| myfitnesspal.com | 483 | beauté test | 7 |
| edsupportforum.com | 307 | madmoizelle.com | 5 |
| shroomery.org | 276 | Magic maman | 5 |
| backyardchickens.com | 264 | boutiqueplaisir.com | 4 |
| somethingawful.com | 205 | Forum ados/public.fr | 4 |
| sparkpeople.com | 190 | psychologies | 4 |
| disqus.com | 172 | aqualiment.com | 3 |
| homedistiller.org | 119 | canardpc.com | 3 |
| lipstickalley.com | 119 | dreadfrance.fr | 3 |
| livestrong.com | 119 | carenity.com | 2 |
| 4chan.org | 117 | courseapied.net | 2 |
| proboards.com | 117 | europepizza.fr | 2 |
| raypeatforum.com | 114 | federation-pizzaiolos-france.fr | 2 |
| nutritionfacts.org | 111 | vegetarisme.fr | 2 |
| homebrewtalk.com | 110 | Yabiladi | 2 |
| youbemom.com | 98 | allocine.fr | 1 |
| homebrewersassociation.org | 92 | audiofanzine.com | 1 |
| freeforums.net | 80 | beaute-addict.com | 1 |
| fark.com | 72 | boursorama.com | 1 |
| godlikeproductions.com | 72 | buveurs-detiquettes.fr | 1 |
| city-data.com | 71 | caradisiac.com | 1 |
| bodybuilding.com | 67 | dailymotion.com | 1 |
| cowboyszone.com | 66 | dealabs.com | 1 |
| yahoo.com | 65 | filae.com | 1 |
| drmcdougall.com | 61 | free.fr | 1 |
| patient.info | 60 | international-hairlossforum | 1 |
| survivalistboards.com | 57 | Journal des femmes | 1 |
| dcurbanmom.com | 48 | kikourou.net | 1 |
| makeupalley.com | 48 | lappart-des-spasmos.fr | 1 |
| democraticunderground.com | 46 | lesimpatientes | 1 |
| healing well | 46 | nutrivi.fr | 1 |
| babycenter.com.au | 44 | otaku-attitude.net | 1 |
| medhelp.org | 42 | pharmabolix.com | 1 |
| babycenter.ca | 40 | raidlight.com | 1 |
| forensicscommunity.com | 39 | rockiemag.com | 1 |
| longhaircommunity.com | 38 | ruche-apiculture.com | 1 |
| qvc.com | 38 | tabledescalories.com | 1 |
| azbaja.com | 37 | touteslesbieres.fr | 1 |
| pizzamaking.com | 36 |  |  |
| neogaf.com | 35 |  |  |
| sportsnstuff.com | 35 |  |  |
| timebomb2000.com | 35 |  |  |
| boards.net | 33 |  |  |
| ycombinator.com | 32 |  |  |
| resetera.com | 31 |  |  |
| the-avocado.org | 31 |  |  |
| flickr.com | 30 |  |  |
| kiwifarms.net | 30 |  |  |
| straightdope.com | 30 |  |  |
| breast cancer | 29 |  |  |
| mrmoneymustache.com | 29 |  |  |
| winemakingtalk.com | 29 |  |  |
| steamcommunity.com | 28 |  |  |
| steemit.com | 28 |  |  |
| mtbr.com | 26 |  |  |
| lowcarber.org | 25 |  |  |
| wonkette.com | 25 |  |  |
| justanswer.com | 24 |  |  |
| nih.gov | 24 |  |  |
| wordpress.com | 24 |  |  |
| fool.com | 23 |  |  |
| google.com | 23 |  |  |
| tumblr.com | 23 |  |  |
| blazblue.com | 22 |  |  |
| gamespot.com | 22 |  |  |
| slickdeals.net | 22 |  |  |
| homebrewinguk.com | 21 |  |  |
| advfn.com | 20 |  |  |
| disboards.com | 20 |  |  |
| owner-manuals.com | 20 |  |  |
| celiac.com | 19 |  |  |
| csnbbs.com | 19 |  |  |
| penny-arcade.com | 19 |  |  |
| thencomesfamily.com | 19 |  |  |
| tigerdroppings.com | 19 |  |  |
| yelp.com | 19 |  |  |
| aarp.org | 18 |  |  |
| anbsoft.com | 18 |  |  |
| seniorforums.com | 18 |  |  |
| usmessageboard.com | 18 |  |  |
| acne.org | 17 |  |  |
| gardenweb.com | 17 |  |  |
| grasscity.com | 17 |  |  |
| newagtalk.com | 17 |  |  |
| welltrainedmind.com | 17 |  |  |
| wineberserkers.com | 17 |  |  |
| schizophrenia.com | 16 |  |  |
| autofinanceadvice.com | 15 |  |  |
| personalitycafe.com | 15 |  |  |
| thegoatspot.net | 15 |  |  |
| anime-alberta.org | 14 |  |  |
| chowhound.com | 14 |  |  |
| delphiforums.com | 14 |  |  |
| freerepublic.com | 14 |  |  |
| Health boards | 14 |  |  |
| nature.com | 14 |  |  |
| overgrow.com | 14 |  |  |
| plantedtank.net | 14 |  |  |
| dendroboard.com | 13 |  |  |
| diabetesdaily.com | 13 |  |  |
| dlisted.com | 13 |  |  |
| forresthealth.com | 13 |  |  |
| freespeechextremist.com | 13 |  |  |
| hotukdeals.com | 13 |  |  |
| ignboards.com | 13 |  |  |
| longecity.org | 13 |  |  |
| mobilism.org | 13 |  |  |
| obesityhelp.com | 13 |  |  |
| worldwebs.com | 13 |  |  |
| foodbanter.com | 12 |  |  |
| gossiprocks.com | 12 |  |  |
| northernbrewer.com | 12 |  |  |
| videofitness.com | 12 |  |  |
| ar15.com | 11 |  |  |
| bariatricpal.com | 11 |  |  |
| hipinion.com | 11 |  |  |
| jackkruse.com | 11 |  |  |
| anabolicminds.com | 10 |  |  |
| chiefsplanet.com | 10 |  |  |
| monstergirl.net | 10 |  |  |
| professionalmuscle.com | 10 |  |  |
| shacknews.com | 10 |  |  |
| texashuntingforum.com | 10 |  |  |
| thebump.com | 10 |  |  |
| thedomainfo.com | 10 |  |  |
| amazon.com | 9 |  |  |
| avianavenue.com | 9 |  |  |
| earlyretirementextreme.com | 9 |  |  |
| gethealthcarerelief.com | 9 |  |  |
| merchantcircle.com | 9 |  |  |
| metafilter.com | 9 |  |  |
| mydukandiary.com | 9 |  |  |
| stagram.com | 9 |  |  |
| bikeforums.net | 8 |  |  |
| Bluelight | 8 |  |  |
| boxden.com | 8 |  |  |
| diychatroom.com | 8 |  |  |
| eggheadforum.com | 8 |  |  |
| eschatonblog.com | 8 |  |  |
| gab.com | 8 |  |  |
| getbig.com | 8 |  |  |
| hefeiexpat.com | 8 |  |  |
| literotica.com | 8 |  |  |
| purseblog.com | 8 |  |  |
| ramsondemand.com | 8 |  |  |
| Social Anxiety Forum | 8 |  |  |
| styleforum.net | 8 |  |  |
| sweat.com | 8 |  |  |
| thegatewaypundit.com | 8 |  |  |
| thespruceeats.com | 8 |  |  |
| tripadvisor.com | 8 |  |  |
| washingtonpost.com | 8 |  |  |
| westlife.org | 8 |  |  |
| whole30.com | 8 |  |  |
| wikidoc.org | 8 |  |  |
| beesource.com | 7 |  |  |
| benzobuddies.org | 7 |  |  |
| deviantart.com | 7 |  |  |
| explore.org | 7 |  |  |
| germanshepherds.com | 7 |  |  |
| hungryonion.org | 7 |  |  |
| msn.com | 7 |  |  |
| not606.com | 7 |  |  |
| parrotforums.com | 7 |  |  |
| poodleforum.com | 7 |  |  |
| sephora.com | 7 |  |  |
| stephanierct.com | 7 |  |  |
| talkaboutmarriage.com | 7 |  |  |
| thehackersparadise.com | 7 |  |  |
| ttlink.com | 7 |  |  |
| able2know.org | 6 |  |  |
| asm.org | 6 |  |  |
| badgerandblade.com | 6 |  |  |
| bobistheoilguy.com | 6 |  |  |
| britishexpats.com | 6 |  |  |
| cjb.net | 6 |  |  |
| ebay.com | 6 |  |  |
| healthunlocked.com | 6 |  |  |
| houzz.com | 6 |  |  |
| hvac-talk.com | 6 |  |  |
| imamother.com | 6 |  |  |
| lunaticoutpost.com | 6 |  |  |
| realgm.com | 6 |  |  |
| rpg.net | 6 |  |  |
| sigforum.com | 6 |  |  |
| susans.org | 6 |  |  |
| thesims.com | 6 |  |  |
| wdwmagic.com | 6 |  |  |
| 1addicts.com | 5 |  |  |
| bimmerpost.com | 5 |  |  |
| brewnosers.org | 5 |  |  |
| digitalspy.com | 5 |  |  |
| dpreview.com | 5 |  |  |
| e90post.com | 5 |  |  |
| eventbrite.com | 5 |  |  |
| frugalvillage.com | 5 |  |  |
| goldenretrieverforum.com | 5 |  |  |
| groupthink2.blogspot.com | 5 |  |  |
| hipforums.com | 5 |  |  |
| instapundit.com | 5 |  |  |
| instructables.com | 5 |  |  |
| investorvillage.com | 5 |  |  |
| m3post.com | 5 |  |  |
| mmo-champion.com | 5 |  |  |
| myjournals.org | 5 |  |  |
| notalwaysright.com | 5 |  |  |
| palmerreport.com | 5 |  |  |
| phook.net | 5 |  |  |
| photographyreview.com | 5 |  |  |
| ramforumz.com | 5 |  |  |
| rawstory.com | 5 |  |  |
| reef2reef.com | 5 |  |  |
| rivals.com | 5 |  |  |
| rollitup.org | 5 |  |  |
| scducks.com | 5 |  |  |
| soberrecovery.com | 5 |  |  |
| talkbass.com | 5 |  |  |
| texags.com | 5 |  |  |
| thehotpepper.com | 5 |  |  |
| thepaceline.net | 5 |  |  |
| veggieboards.com | 5 |  |  |
| xbimmers.com | 5 |  |  |
| 2addicts.com | 4 |  |  |
| 3fatchicks.com | 4 |  |  |
| 5post.com | 4 |  |  |
| 8ch.net | 4 |  |  |
| alcoholicpoet.com | 4 |  |  |
| archive.org | 4 |  |  |
| arstechnica.com | 4 |  |  |
| beersmith.com | 4 |  |  |
| bitchute.com | 4 |  |  |
| bogleheads.org | 4 |  |  |
| brighteon.com | 4 |  |  |
| cheftalk.com | 4 |  |  |
| cheyennejournal.com | 4 |  |  |
| collegeconfidential.com | 4 |  |  |
| dailystrength.org | 4 |  |  |
| daxueyingyu.com | 4 |  |  |
| diybeer.com | 4 |  |  |
| early-retirement.org | 4 |  |  |
| expatriates.com | 4 |  |  |
| freelancer.com | 4 |  |  |
| ibtimes.com | 4 |  |  |
| icmag.com | 4 |  |  |
| ign.com | 4 |  |  |
| macrumors.com | 4 |  |  |
| mandatory.com | 4 |  |  |
| manrepeller.com | 4 |  |  |
| michellesmirror.com | 4 |  |  |
| pickuplimes.com | 4 |  |  |
| sidexsideworld.com | 4 |  |  |
| simplelivingforum.net | 4 |  |  |
| spacebattles.com | 4 |  |  |
| stormfront.org | 4 |  |  |
| studentdoctor.net | 4 |  |  |
| theapricity.com | 4 |  |  |
| thehulltruth.com | 4 |  |  |
| treato.com | 4 |  |  |
| uk-muscle.co.uk | 4 |  |  |
| vapingunderground.com | 4 |  |  |
| vietfun.com | 4 |  |  |
| vipmembervault.com | 4 |  |  |
| vitamarket.net | 4 |  |  |
| worldstarhiphop.com | 4 |  |  |
| wowdigsite.com | 4 |  |  |
| xoxohth.com | 4 |  |  |
| absolutewrite.com | 3 |  |  |
| adiforums.com | 3 |  |  |
| advrider.com | 3 |  |  |
| anandtech.com | 3 |  |  |
| answers.com | 3 |  |  |
| automaticwasher.org | 3 |  |  |
| biology-forums.com | 3 |  |  |
| blizzard.com | 3 |  |  |
| bonanza.com | 3 |  |  |
| botanicchoice.com | 3 |  |  |
| butchfemmeplanet.com | 3 |  |  |
| camerareadycosmetics.com | 3 |  |  |
| christianforums.com | 3 |  |  |
| crsociety.org | 3 |  |  |
| curezone.org | 3 |  |  |
| cyclingnews.com | 3 |  |  |
| dailykos.com | 3 |  |  |
| diabetesforum.com | 3 |  |  |
| e-cigarette-forum.com | 3 |  |  |
| edmunds.com | 3 |  |  |
| ehealthforum.com | 3 |  |  |
| future4200.com | 3 |  |  |
| giffgaff.com | 3 |  |  |
| goodreads.com | 3 |  |  |
| havaneseforum.com | 3 |  |  |
| homesteadingtoday.com | 3 |  |  |
| hotair.com | 3 |  |  |
| hystersisters.com | 3 |  |  |
| indusladies.com | 3 |  |  |
| joemygod.com | 3 |  |  |
| lawyersgunsmoneyblog.com | 3 |  |  |
| macresource.com | 3 |  |  |
| masterofmalt.com | 3 |  |  |
| misshaus.com | 3 |  |  |
| mrbeer.com | 3 |  |  |
| myanimelist.net | 3 |  |  |
| mysupportforums.org | 3 |  |  |
| opeforum.com | 3 |  |  |
| openkore.com | 3 |  |  |
| pinterest.com | 3 |  |  |
| plantbasedonabudget.com | 3 |  |  |
| primetimer.com | 3 |  |  |
| priuschat.com | 3 |  |  |
| quartertothree.com | 3 |  |  |
| recipebridge.com | 3 |  |  |
| religiousforums.com | 3 |  |  |
| sciencebasedmedicine.org | 3 |  |  |
| siliconinvestor.com | 3 |  |  |
| sjogrensworld.org | 3 |  |  |
| skyscrapercity.com | 3 |  |  |
| slapmagazine.com | 3 |  |  |
| sphynxlair.com | 3 |  |  |
| supportgroups.com | 3 |  |  |
| swolesource.com | 3 |  |  |
| tdpri.com | 3 |  |  |
| timekeeperforum.com | 3 |  |  |
| topix.com | 3 |  |  |
| tortoiseforum.org | 3 |  |  |
| twoplustwo.com | 3 |  |  |
| veganessentials.com | 3 |  |  |
| viesearch.com | 3 |  |  |
| windsorpeak.com | 3 |  |  |
| woodenboat.com | 3 |  |  |
| yellowbullet.com | 3 |  |  |
| Others | 657 |  |  |
