# Supplementary material for: Natural Language Processing and Machine Learning Techniques for Analyzing Conversations About Nutritional Yeasts in the United States and France: Retrospective Social Media Listening Study
Source: JMIR Infodemiology. 2025 May 1;5:e60528. doi: 10.2196/60528 (PMC12061346; doi:10.2196/60528)
Supplement: Multimedia Appendix 3 [file infodemiology-v5-e60528-s003.docx]

**Multimedia Appendix 2: Examples of messages in English and French**

| **Example of message in English** | **Example of message in French** |
| --- | --- |
| **US** | |
| *«  Yesterday's lunch was a salty (and spicy ... Def went too far on the red bell pepper 😳) and hit the spot! Nutritional yeast may sound a bit odd, but it's a great source of B vitamins for eaters »* | “Le déjeuner d'hier était salé (et épicé... j'ai vraiment trop mis de poivron rouge 😳) et c'était exactement ce qu'il me fallait ! La levure nutritionnelle peut sembler un peu étrange, mais c'est une excellente source de vitamines B pour les consommateurs.” |
| *«I bought a big carton of egg whites, what I did with the tofu scrabble was to add nutritional yeast, turmeric for color and black pepper, which makes it taste whole and with whole egg and egg white too. »* | “J'ai acheté un grand carton de blancs d'œufs. Ce que j'ai fait avec le tofu brouillé, c'est d'ajouter de la levure nutritionnelle, du curcuma pour la couleur et du poivre noir, ce qui donne un goût complet, comme avec un œuf entier et du blanc d'œuf aussi.” |
| **France** | |
| *“personally, brewer's yeast worked really well, no side effects, I also tried oenobiol, which wasn't bad, and now I'm starting forcapil, apparently it's a wonder I can't wait to have Rapunzel's hair.”* | “perso la levure de bière a super bien marché , pas d’effets secondaires , j’ai essayé oenobiol aussi pas mal et la je commence forcapil apparemment c’est une dinguerie j’ai hâte d’avoir la chevelure de Raiponce.” |
| *“I used to lose handfuls of them too, that's why they're so damaged, you need to make frequent oil masks that you leave on overnight, rinse them with cold water and take brewer's yeast to strengthen them ☺️ ”* | “Ca me le faisait avant j’en perdait des poignées aussi, c’est prk ils sont cher abîmés, faut que tu te fasses souvent des masques d’huiles que tu laisse la nuit, les rincer a l’eau froide et prendre de la levure de bière pour les fortifier ☺️ ” |
| “*No problem with taking care of oneself with all kinds of oils (jojoba, coconut, castor) natural shampoo like liperol, and brewer's yeast in capsules and powder form (I like the taste)*.” | “ Aucun souci de prendre soin de soi Huile en tout genre (jojoba, coco rincin) Shampoing naturel liperol , gélule et poudre levure de bière (j'aime le goût) ” |
